# The impact of digestion is essential to the understanding of milk as a drug delivery system for poorly water soluble drugs

**DOI:** 10.1016/j.jconrel.2018.10.027

**Published:** 2018-12-28

**Authors:** Ben J. Boyd, Malinda Salim, Andrew J. Clulow, Gisela Ramirez, Anna C. Pham, Adrian Hawley

**Affiliations:** a Drug Delivery, Disposition and Dynamics, Monash Institute of Pharmaceutical Sciences, Monash University (Parkville Campus), 381 Royal Parade, Parkville, VIC 3052, Australia; b ARC Centre of Excellence in Convergent Bio-Nano Science and Technology, Monash Institute of Pharmaceutical Sciences, Monash University (Parkville Campus), 381 Royal Parade, Parkville, VIC 3052, Australia; c SAXS/WAXS beamline, Australian Synchrotron, ANSTO, 800 Blackburn Road, Clayton, VIC 3169, Australia

**Keywords:** Milk, Lipid-based formulation, Drug solubilisation, Polymorphism, X-ray scattering, In vitro digestion, Halofantrine, Weakly basic drug

## Abstract

Milk has previously been considered as a potential lipid-based drug delivery system for poorly water soluble drugs but it has never gained significant attention. This is in part because relying on solubility in lipid-based formulations (in this case milk) does not provide a complete picture of the behavior of such systems upon digestion. Herein, we demonstrate using time resolved X-ray scattering that the digestion of milk is actually crucial to the solubilisation of a poorly water-soluble drug, halofantrine. Halofantrine was chosen because its behaviour in lipid-based formulations has been widely investigated and because of its close structural relationship to lumefantrine, an antimalarial drug of current interest for the treatment of paediatric malaria. The transformation of the drug from a crystalline solid form in suspension in milk, to a solubilised form as a direct consequence of lipolysis highlights that consideration of digestion of the milk lipids as a critical process that influences drug solubilisation and availability for absorption is vital.

## Graphical abstract

**Figure:**
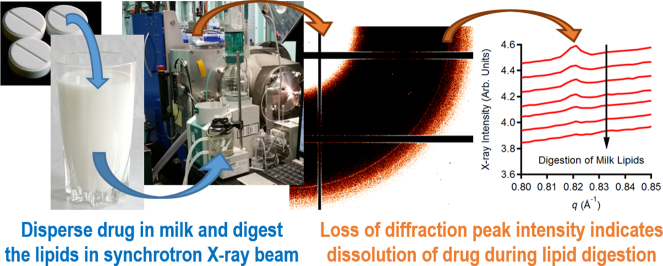

## 1 Introduction

Milk is nature's optimised delivery system – providing sufficient bioavailability of lipids, protein and carbohydrate to sustain life in the critical early months after birth. Despite this realisation, the use of milk in a drug delivery context has not advanced to the point of it being formally considered as an excipient.

The fat content in mammalian milk comprises over 95% triglycerides [1]. The recognition that these milk lipids can offer a solubilising environment for poorly water-soluble lipophilic drugs has led to a range of studies focusing on the solubility of drugs in milk [[2], [3], [4], [5], [6]] and in a limited number of studies the effect of milk on *in vivo* absorption and bioavailability [[7], [8], [9]]. The solubility of drugs in milk has been reported to be enhanced compared to that in aqueous media without fat present and in limited *in vivo* studies consequent increases in systemic exposure have been demonstrated. However, the mechanism by which milk lipids enhance drug solubilisation and absorption has not been fully demonstrated in these studies, despite it being of critical importance to the fate of most Class II drugs according to the Biopharmaceutics Classification System, which constitute the majority of drugs arising from drug discovery programs.

It is well known that the performance of pharmaceutical lipid-based formulations containing triglycerides is critically dependent on digestion of the triglycerides to form monoglycerides and fatty acids [[10], [11], [12]], which form colloidal structures that provide an optimal solubilisation environment [13]. Drugs are often more soluble in the colloidal self-assembled liquid crystalline structures formed by the lipolysis products than they are in the parent amorphous triglycerides. Furthermore, the formation of fatty acids during digestion is increasingly being recognised as an important factor in the solubilisation of weakly basic crystalline compounds [[14], [15], [16]], whereby ion pair formation with the fatty acids liberated upon digestion can facilitate solubilisation of crystalline drug [15,17].

The lipids that constitute milk vary between different species. The major differences between human breast milk and bovine milk (a major part of the Western adult diet) lie in the fatty acid (FA) distribution (there are more shorter chain FAs in cow's milk than human milk) and the nature of the *sn*-2 fatty acids, being typically saturated (e.g., palmitic acid) in human milk and unsaturated (e.g., oleic acid) in cow's milk [18]. The presence of the saturated 2-monoglycerides after digestion of triglycerides in human milk and the interaction of saturated fatty acids with minerals in the gut is important for nutritional and gut health in the infant [19]. Despite these differences in lipid composition, we have recently reported that the digestion of the lipids in milk by lipases induces self-assembly into highly ordered mesophases, which are surprisingly similar between species [20,21]. While the underlying, possibly evolutionary, purpose for this structuring (if there is one) is not yet understood, the digestion-induced structuring of the lipids in the fat droplet is profound and suggests the likely role of milk in other functions than merely presenting lipids in an absorbable form.

**Fig. 1:**
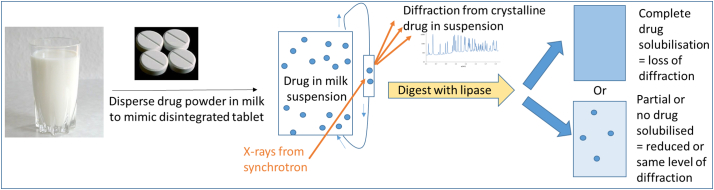
– Concept of using changes in intensity of drug diffraction peaks recorded using time-resolved small angle X-ray scattering to determine the influence of lipid digestion on drug solubilisation during the digestion of milk.

With lipid digestion being so critical to the nutritional outcomes of milk consumption and to the performance of triglyceride-containing lipid-based formulations, it is somewhat surprising that there does not appear to be any focus in the open literature about the dynamic effect of digestion of milk fats on the fate of incorporated poorly water-soluble drugs. There has been one report in the literature of drug equilibrium solubility in pre-digested milk as a medium [22], with interesting correlations obtained that highlight that digestion should be considered when thinking about milk and drug solubilisation. We have recently shown the powerful ability of time-resolved synchrotron X-ray diffraction to elucidate drug precipitation during digestion of lipid-based formulations [23] and polymorphic transformations between different solid state forms of drugs [24]. With these techniques at our disposal we have determined how critical the digestion of milk, and in particular the fat content, is to the fate of co-administered poorly water-soluble drugs using the concept illustrated in Fig. 1.

## 2 Material and methods

### 2.1 Materials

Commercial homogenised and pasteurised full fat bovine milk (3.8 % fat) and low fat milk (1.3% fat) were purchased from a local supermarket (Woolworths Brunswick or Coles Mt. Waverley, Victoria, Australia) or supplied by the Australian Synchrotron and Table 1 details the compositional information. Halofantrine base (>99% purity) was purchased from GlaxoSmithKline (King of Prussia, PA). Trizma maleate (reagent grade) and casein from bovine milk (technical grade) were purchased from Sigma-Aldrich (St. Louis, Missouri). Calcium chloride dihydrate (>99% purity) and sodium hydroxide pellets (min. 97% purity) were purchased from Ajax Finechem (Seven Hills, New South Wales, Australia). Hydrochloric acid (36%) was purchased from LabServ (Ireland). Sodium chloride (>99% purity) was purchased from Chem Supply (Gillman, South Australia, Australia). USP grade pancreatin extract was purchased from Southern Biologicals (Nunawading, Victoria, Australia). All chemicals were used without further purification and water was sourced from a Millipore Milli-Q purification system.

**Table 1:** Table of composition information for full fat and low fat milk used in this study. Pauls-branded milk (Parmalat Australia Pty Ltd., Queensland, Australia) was used for all experiments involving milk.

Nutritional information	Quantity per 100 mL	
	Full fat milk (Pauls milk full cream)	Low fat milk (Pauls Rev low fat milk)
Total fat	3.8 g	1.3 g
Saturated fat	2.5 g	0.8 g
Protein	3.4 g	3.4 g
Carbohydrate (sugars)	4.8 g	4.9 g
Sodium	40 mg	40 mg
Calcium	115 mg	119 mg
Vitamin A	41 μg	39 μg
Riboflavin (Vitamin B2)	0.2 mg	N/A
Vitamin D	N/A	0.5 μg

## 3 Methods

### 3.1 Preparation of halofantrine suspension in milk, in vitro lipolysis and scattering measurements

The *in vitro* lipolysis apparatus and its coupling to the SAXS/WAXS beamline at the Australian Synchrotron [25] (as shown schematically in Fig. 1) have been described in detail previously [23]. Halofantrine base (40 mg) was mixed with fasted intestinal media (5 mL, containing 5.44 mM sodium taurodeoxycholate and 1.13 mM 1,2-dioleyl-*sn*-glycero-3-phosphatidylcholine dissolved in Tris buffer as described previously [26]) to provide a coarse suspension for mixing with milk (20 mL). To simulate a gastric step, the halofantrine base was treated with water (2.50 mL) and 1 M HCl (0.25 mL) prior to the addition of fasted intestinal media (5 mL). The suspension was added to the thermostatted (37 °C) glass vessel under constant magnetic stirring. The pH of each sample was adjusted to 6.500 ± 0.005 prior to the start of each measurement and scattering data acquisition commenced remotely. An X-ray beam with a wavelength *λ* of 0.954 Å (photon energy = 13 keV) was used with a sample-to-detector distance of approximately 0.6 m to afford a *q* range of 0.04 < *q* (Å^–1^)< 2.00. *q* is the length of the scattering vector defined by (4π/*λ*)sin(*2θ*/2), where *2θ* is the scattering angle. The diffraction patterns were recorded using a Pilatus 1 M detector with a 5 s acquisition time and a delay of 15 s between measurements (one measurement every 20 s). The raw data were reduced to 1D scattering functions I(*q*) by radial integration using the in-house-developed software ScatterBrain.

After stirring and acquiring background diffraction of the suspended crystalline drug for several min, pancreatin suspension (~1000 TBU per mL of digest) was remotely injected into the vessel to initiate lipolysis. The pH of the sample in the digestion vessel was maintained at 6.5 during digestion using either 2.0 M or 0.6 M aqueous sodium hydroxide solution titrated in by a pH stat control module (Metrohm AG, Herisau, Switzerland). Tris-maleate buffer (50 mM, pH 6.5) was used for the digestions and was prepared by dissolving 11.86 g of Trizma-maleate, 0.74 g of calcium chloride dihydrate (5 mM), and 8.77 g of sodium chloride (150 mM) in water to a total volume of 1000 mL. Calcium chloride was added to remove the free fatty acids from the lipolysis medium that could inhibit lipolysis and to more closely resemble *in vivo* conditions where the free fatty acids are absorbed [3]. The pH stat component of the lipolysis model provides an indication of the kinetics of the lipolysis reaction by monitoring the addition of sodium hydroxide in response to production of the fatty acids to maintain a constant pH (6.5 in these studies).

## 4 Results and discussion

Halofantrine free base was dispersed in full fat milk (3.8% fat, see Table 1 for more detail), low fat milk (1.3% fat) or milk protein (casein) solution in tris buffer (no fat) as a suspension (40 mg in 20 mL of vehicle) together with 5 mL of simulated fasted intestinal media [27], and solubilisation of the suspended crystalline drug was monitored through the disappearance of the diffraction peaks associated with halofantrine crystals during digestion. Halofantrine has a significantly greater solubility in fatty acids than in the corresponding undigested triglycerides as is common for many poorly soluble, weakly basic drugs [28]. Thus, in the case of long chain triglycerides (i.e. the major component of the fats in milk) digestion was expected to stimulate solubilisation of the drug from the solid crystalline state as a consequence of the production of fatty acids.

**Fig. 2:**
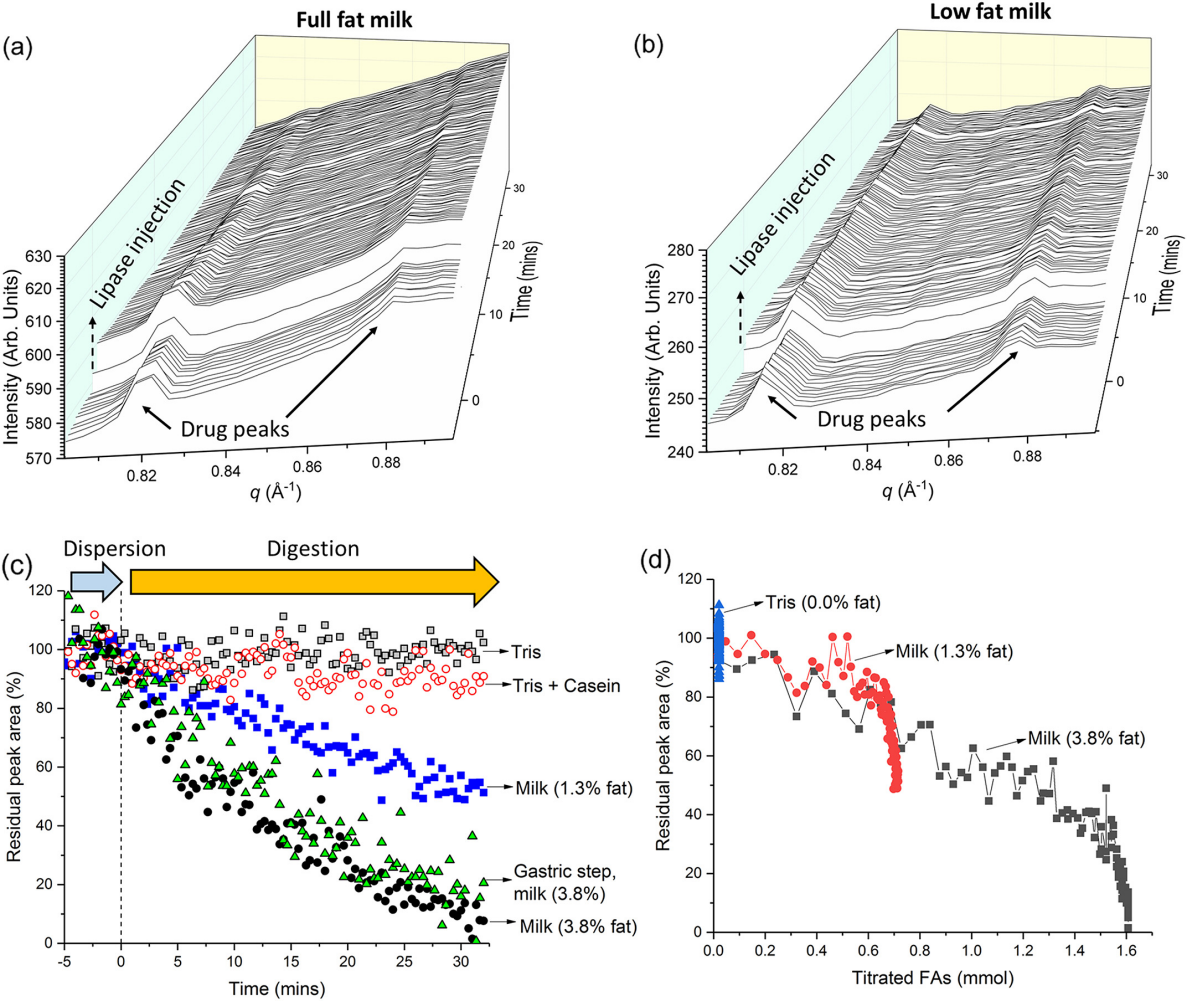
– Panels (a) and (b) Diffraction profiles of halofantrine free base suspended in full fat milk and low fat milk respectively as a function of time prior to and after addition of pancreatin extract. Panel (c) Residual integrated peak area of the characteristic diffraction peak for halofantrine at *q* = 0.82 Å^−1^ in full and low fat milk derived from the profiles in Panels (a) and (b) and the corresponding areas observed during digestion in low fat milk, tris buffer, tris buffer containing casein, and full fat milk after a gastric acidification step. Panel (d) illustrates the residual crystalline drug [determined from the area of the diffraction peak at *q* = 0.82 Å^−1^ in Fig. 2 (c)] as a function of the titratable fatty acids produced during digestion.

Fig. 2, Panel (a) illustrates that prior to the initiation of lipolysis (in this format by remote-controlled injection of pancreatin extract), crystalline drug was present as a suspension in the circulating formulation through the flow capillary, which gives rise to the drug diffraction peaks at early times. The injection of lipase stimulates a clear decrease in the intensity of the diffraction peaks corresponding to the crystalline drug present, with the diffraction peaks for drug in full fat milk vanishing completely after approximately 30 min. It should be noted that there is no alternative method currently available to obtain the same time-resolved information about the fate of crystalline drugs during lipolysis and that the synchrotron X-ray source is crucial to enabling the kinetic resolution to follow this process in real time at such low dosage-relevant concentrations of crystalline drug (~0.2% *w/v*).

By tracking the peak area of one representative diffraction peak in the diffractogram (the peak at *q* = 0.82 Å^−1^), the kinetics of solubilisation could be determined and this illustrates the transformation from drug in crystalline form to a solubilised form [Fig. 2, Panel (c)]. It is even clearer from this representation that the fat content of the milk and digestion together are crucial to the solubilisation process. The raw diffraction data for halofantrine in low fat milk are presented in Fig. 2 panel (b). The use of low fat milk did not decrease the intensity of the drug diffraction peak to the same extent as full fat milk (i.e., some drug remained dispersed in crystalline form) and the use of either buffer or buffer containing a relevant proportion of casein to mimic the total protein in milk did not provide any significant drug solubilisation because of the absence of lipid digestion products. Furthermore, a gastric step prior to the commencement of intestinal lipolysis during which the drug was acidified was also performed and demonstrated the lack of impact of transiting this condition to the overall fate of the drug upon intestinal lipolysis.

**Fig. 3:**
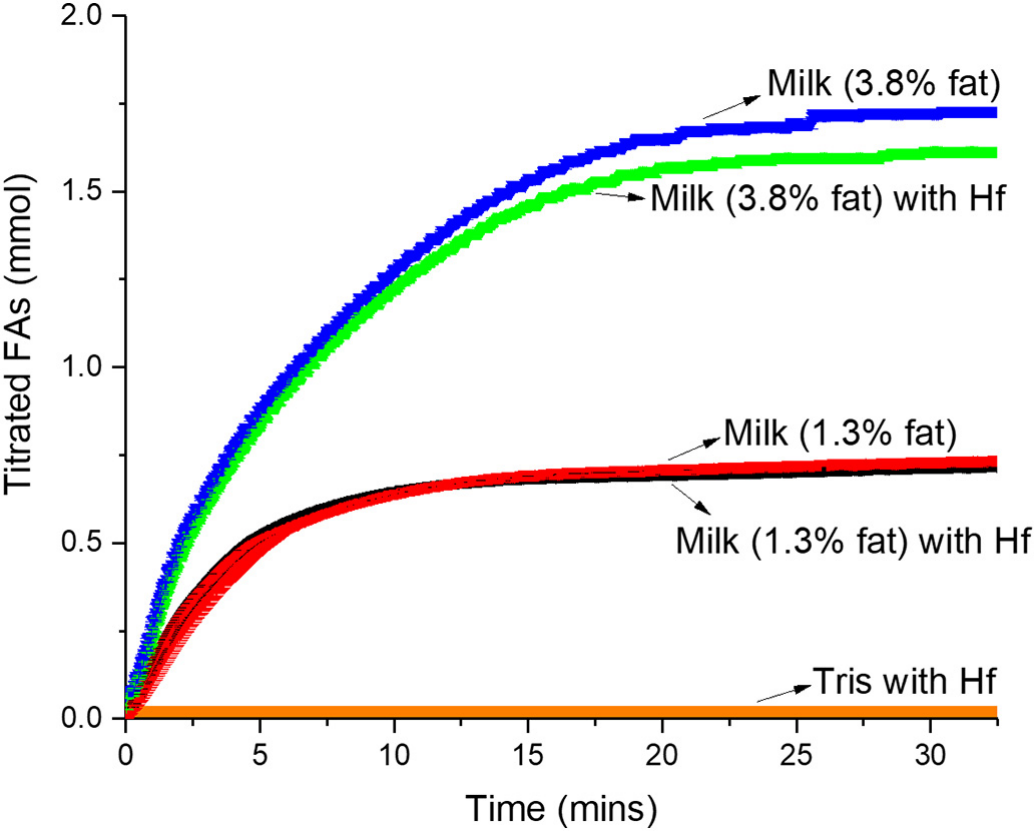
– Titration profile showing the millimolar rate of addition of sodium hydroxide to digesting full fat milk, low fat milk and buffer containing no milk fat in the absence and presence of drug (Hf) to maintain the pH at 6.5. All milk titration profiles are the average of three independent digestion measurements with associated error bars (= standard deviation) and the buffer only (Tris) digestion was performed only once.

The kinetics of the digestion process were obtained by measuring the titration of liberated fatty acids by the pH stat system with sodium hydroxide (Fig. 3), which allows transformation of the time dependent data in Fig. 2 (c) into the amount of crystalline drug present as a function of the extent of lipolysis in Fig. 2 (d). In Fig. 2 (d) there is a clear and approximately linear decrease in crystalline drug content with fatty acid production throughout most of the digestion process, thereby highlighting the importance of lipid digestion to the fate of the poorly water-soluble drug during the digestion of milk.

The ion pairing of weakly basic drugs with fatty acids formed upon digestion of lipids is currently receiving significant interest [[15], [16], [17]], in part because there is debate about the importance of the solid state form of drug in dictating absorption but also because it may emerge as a major factor in providing a food effect. Understanding the interaction between digested lipids and drugs in the case of lipid formulations, and milk as shown here, would therefore appear to be of much greater importance to optimising performance than the solubility of drug in the initial formulation/milk.

Development of products using milk still remains a major hurdle [29]. Although spray dried milk powder and freeze dried milk [30,31] have been previously studied as vehicles for poorly water-soluble drugs, albeit in a dissolution context, their use has not progressed into products. The inherent variability in the composition of milk and milk powders makes the regulatory hurdle for adoption of milk fat components as pharmaceutical excipients challenging compared to more well-defined lipid excipients. Nevertheless, milk has long been used extemporaneously as a vehicle for paediatric formulations and as there is a benefit in controlling the fat to drug levels in lipid-based medicines, the use of milk powder as a formal excipient is therefore expected to gain interest. Milk powder has a particular advantage in enabling solid dosage forms [32], whilst simultaneously affording control over the lipid content.

## 5 Conclusion

This work has shown the impact of the *digestion* of milk on the fate of a co-administered poorly water-soluble drug, highlighting the necessity of digestion and fatty acid production in the solubilisation of drug. Control measurements confirmed the absence of an effect due to protein. The findings demonstrate the necessity for further study of this phenomenon to advance the case for the use of milk fats as excipients through increased understanding of the role of digesting milk lipids on the solubilisation of drugs under intestinal conditions. While milk in its entirety would face significant challenges in a regulatory sense there are opportunities for formulation specifically with the major triglycerides constituting milk, or highly regulated milk powders to potentially enable development of products based on these concepts. Unlocking the mechanism by which milk can enhance drug solubilisation is a necessary first step towards determining a path to regulatory approval of excipients that operate by a similar mechanism.

## Funding sources

Bill & Melinda Gates Foundation (Investment ID OPP1160404).

Australian Research Council (DP160102906).

## Conflict of interest

The authors declare no competing financial interest.
